# Stray Light Analysis and Suppression of the Visible to Terahertz Integrated Cloud Detection Optical System

**DOI:** 10.3390/s23084115

**Published:** 2023-04-19

**Authors:** Haiwei Jiang, Xinhua Niu

**Affiliations:** 1 Key Laboratory of Infrared System Detection and Imaging Technology, Chinese Academy of Sciences, Shanghai 200083, China; jianghaiwei18@mails.ucas.ac.cn; 2 University of Chinese Academy of Sciences, Beijing 100049, China; 3 Shanghai Institute of Technical Physics, Chinese Academy of Sciences, Shanghai 200083, China

**Keywords:** stray light suppression, terahertz, infrared, cloud detection

## Abstract

The wide-spectrum integrated imaging method can simultaneously obtain the spectral information of different spectral bands of the same target, which is conducive to the realization of the high-precision detection of target characteristics, and can simultaneously obtain more comprehensive elements such as the structure, shape, and microphysical parameters of the cloud. However, for stray light, the same surface has different characteristics at different wavelengths, and a wider spectral band means more complex and diverse sources of stray light, which renders the analysis and suppression of stray light more difficult. In this work, according to the characteristics of the visible-to-terahertz integrated optical system design scheme, the influence of material surface treatment on stray light was studied; the stray light analysis and optimization of the whole link of light transmission were carried out. For the sources of stray light in different channels, targeted suppression measures such as front baffle, field stop, special structure baffle, and reflective inner baffle were adopted. The simulation results indicate that when the off-axis field of view was greater than 10°. The point source transmittance (PST) of the terahertz channel is on the order of 10^−4^, the visible and infrared channels are less than 10^−5^, and the final terahertz PST was on the order of 10^−8^, while visible and infrared channels were lower than 10^−11^. Here, we present a method for stray light suppression based on conventional surface treatments for broadband imaging systems.

## 1. Introduction

Clouds are one of the important factors affecting the global climate; they play a vital role in the energy budget of the atmosphere and the hydrological cycle. However, due to the lack of more comprehensive cloud observation data, general circulation models are not accurate enough, which further affects the accuracy of weather forecasts and disaster warnings. Improving cloud observation capabilities could help solve these problems [1,2].

At present, the main passive cloud satellite-borne remote-sensing instruments are mostly in visible (VIS) and infrared (IR) spectral ranges. Most meteorological satellites in various countries carry cloud passive optical detection payloads for cloud parameter observation, such as the Visible Infrared Imaging Radiometer Suite (VIIRS) of the United States; the Meteorological Imager (MET image) of the European Space Agency; the Medium Resolution Spectral Imager (MERSI) carried by China’s Fengyun-3 series of meteorological satellites, etc. [3,4,5]. For ice clouds, the particle size is approximately concentrated in the range of 20–600 μm [6]. VIS and IR sensors are not sensitive enough to detect ice clouds, but the terahertz (THz) wavelength is equivalent to this range. Combining VIS and IR can not only achieve a more comprehensive range of ice cloud detection but also realize the mutual complementary support of data information, making the observation data more accurate and perfect. The United States, Europe, and other countries have already carried out research on ice cloud measurements in the THz spectral range. Europe’s next-generation meteorological satellite could also carry a THz ice cloud imager [7,8].

Due to the particularity of THz in the position of the electromagnetic spectrum, THz passive imaging is realized both electrically and optically. For example, the microwave limb sounder (MLS) carried by the Aura satellite adopts the electrical THz imaging method and realizes THz detection through the heterodyne method [9]. Optical THz detection is realized by an optical method similar to that of VIS and IR. There are both transmissive and reflective structures. The transmissive structure is limited by the lens material and weight. Therefore, it is not suitable for large-aperture optical systems. For example, The Diviner Lunar Radiometer Experiment carried by NASA’s Lunar Reconnaissance Orbiter (LRO) has a detection wavelength range of 0.35–400 μm, and the system adopts an off-axis three-mirror structure [10,11].

VIS and IR optical sensors have been developed for a long time, and their stray light analysis and suppression technologies are relatively mature [12,13]. As a passive thermal detection, THz also has the problem of its own thermal radiation background. Most astronomical observation instruments in the THz spectral range have high requirements for stray light suppression and generally adopt the cooling method of the whole machine to reduce their own background radiation. The Herschel Space Observatory (HSO) uses liquid helium refrigeration to make the whole machine work at a temperature of 85 K to reduce the influence of stray light [14]. However, there are few THz optical instruments for earth observation, so the report of the analysis and suppression of stray light in the THz spectral range has not been seen yet. This paper took a VIS, IR, and THz integrated broadband optical system as the object, and focused on the stray light analysis and suppression methods research for the THz channel.

## 2. Parameters of Optical System

In order to realize the detection of a wider spectrum, the system adopts the integrated imaging method of VIS, IR, and THz. Broad-spectrum imaging can obtain more comprehensive cloud information elements, while integrated imaging can simultaneously obtain the spectral information of different channels of the cloud. This method is more comprehensive, and can simultaneously obtain the VIS, IR, and THz spectral information of the same cloud target, which can provide more convenient and efficient cloud observation data for solving the problem of the limited precision caused by imperfect cloud parameters in current general circulation models.

The imager detects spectral information of 10 channels in total, including four visible and near-infrared (VNIR); two short-wave infrared (SWIR); three thermal infrared (TIR); and one terahertz (THz) channel. VNIR and SWIR channels can be used to detect cloud optical thickness and effective particle radius, and TIR channels as the detection channel of the cloud top temperature and cloud phase state [15,16]. THz channel is used to detect ice clouds and the ice water path (IWP). The channel is one of the ideal ice cloud detection channels; it is the most sensitive to ice clouds [17,18,19]. The imager’s main design indicators are shown in Table 1.

**Figure 1:**
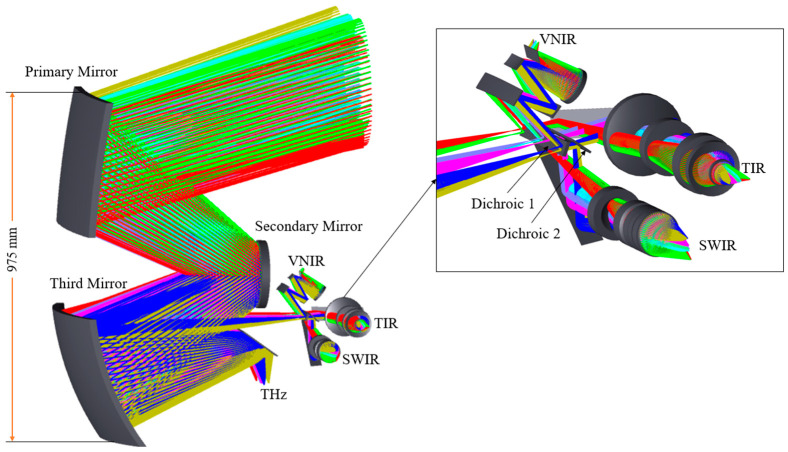
Overall diagram of the optical system.

Due to the long detection wavelength span, the system adopts secondary imaging. The front telescope adopts an off-axis three-mirror anastigmatic (TMA) structure, and the front TMA telescope directly images the terahertz channel [20]. After the VIS and IR channels pass through the front TMA telescope, it is imaged again through the respective rear optical paths. The VIS and IR channels are separated with a THz channel through the field of view. Then, the channels are separated by the beam splitter [21]. The system structure is shown in Figure 1.

Each channel of this system requires different apertures, so the apertures are separately set. At the same time, due to the large field of view, the front telescope adopts a non-re-imaging TMA structure. Compared with the re-imaging TMA structure, the stray light suppression ability is weak [22].

In addition, because of the wide wavelength span it detects, the surface of the structure has different optical properties at different wavelengths. Not only do we need to understand the broad-spectrum characteristics of materials, but we also need to take specific stray light suppression measures for different characteristics of different bands. 

## 3. Stray Light Analysis and Suppression

Stray light can be divided into three categories according to different sources: stray light outside the field of view, stray light inside the field of view, and internal radiation stray light [23,24]. Stray light outside the field of view is non-imaging light that enters the optical system from strong external radiation sources such as the sun, the Earth’s atmosphere, and the moon, and then enters the detector through internal structure scattering and reflection. The stray light in the field of view is caused by the residual reflection on the surface of the optical element, which causes part of the imaging light to enter the system and reach the image plane through an abnormal optical path, that is, the abnormal propagation of the imaging light. The internal radiation stray light is caused by the thermal radiation of the system, including optical components or mechanical surfaces themselves.

Stray light damages the imaging quality of the system, reduces the signal-to-noise ratio, and even directly irradiates the detector, resulting in a decrease in the imaging quality and even the saturation of the detector [25].

The suppression of stray light can be realized by setting main baffles, field stop, or setting a baffle at a special position. The change in surface properties, such as increasing the surface roughness, and increasing the absorption capacity of the material to the detection wavelength, can also be an effective means to eliminate stray light. However, increasing surface absorptivity also means higher surface emissivity, which needs to be carefully considered for thermal infrared and terahertz channels. The suppression of background radiation can be achieved by reducing the temperature of the system or critical surface [26].

Compared with the VIS and IR spectral range, the THz has some different characteristics.In the case of normal incidence, the total reflection of the electromagnetic wave by the surface can be expressed as the sum of specular reflectance and diffuse reflectance [27]:

(1) $$R_{0}=R_{s}+R_{d},$$

Further, $R_{s}$ and $R_{d}$ can be expressed as

(2) $$R_{s}=R_{0}\exp[{\left(-4\pi \frac{\sigma }{\lambda }\right)}^{2}],$$

(3) $$R_{d}=R_{0}\{1-\exp[{\left(-4\pi \frac{\sigma }{\lambda }\right)}^{2}]\},$$

where $R_{0}$ represents the total reflectance, and $R_{s}$ and $R_{d}$ represent the specular reflectance and diffuse reflectance, respectively. σ is the Surface roughness and λ is the incident wavelength. The diffuse reflectance of the component to the incident radiation is affected by the ratio of its surface roughness to the incident wavelength. The smaller the ratio of the two, the smaller the diffuse reflectance it shows. Compared with VIS and IR, under the same surface roughness, the THz wavelength is longer, and the surface of the component has a more specular reflectance [28].

In addition, the common blackened coating will have a large decrease in the absorption in the THz spectral range, which will increase the reflectance. These differences make stray light more difficult for THz channels than for VIS and IR.

Finally, the phenomenon of spontaneous emission on the surface of the structure will also affect the THz channel. Careful consideration should be given to the treatment of the surface of the structural part. While suppressing external stray light, it is necessary to reduce the influence of self-radiation on the THz channel.

### 3.1. Surface Treatment and Reflectivity Characteristics of Structural Parts

For the stray light analysis of broadband imaging systems, understanding the broadband optical absorption characteristics of structural materials is a prerequisite.

There have been many studies on the optical properties of blackened materials in the THz spectral range. As early as the 1880s, NASA studied changes in the reflectivity of common black paint in the IR to THz (40~425 μm) before and after long-term exposure in space. The results show that under long-term space exposure, the reflectance of the coating almost decreased, especially in the far-infrared [29]. Fang et al. mixed silicon carbide particles with 3M black paint to create a high-absorbing THz coating, and the reflectivity of the coating could be less than 0.01 [30]. Since the coating was developed for the THz, no VIS and IR reflectance test results have been seen.

Blackening coatings for the VIS, IR, or THz spectrum alone have been well developed, and the coatings also have good absorptivity. However, the absorbing coating specially developed for the THz or VIS IR spectrum is not necessarily completely suitable for the broadband optical system. Therefore, the conventional surface coating ERB-2 black paint is used as the structural coating.

**Figure 2:**
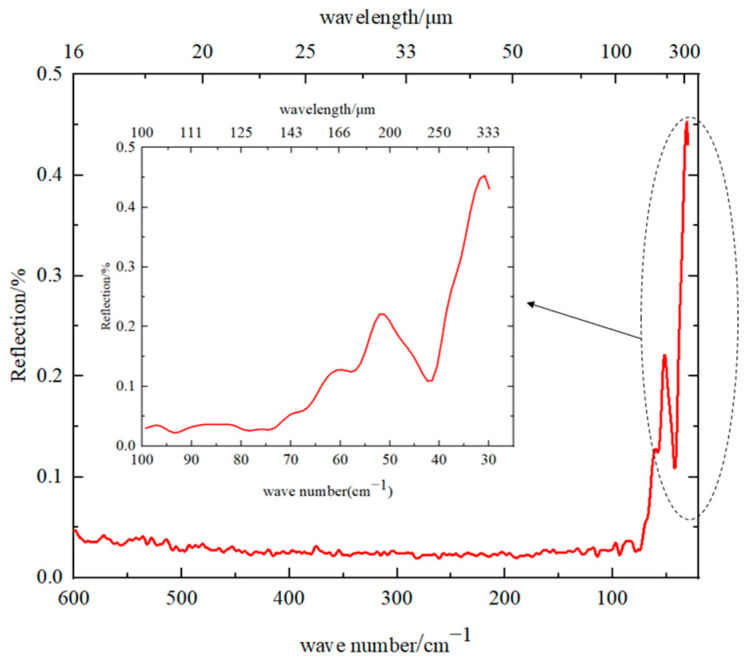
Reflection of ERB-2 paint.

The reflectance of the sample coated with ERB-2 black paint was measured with a Fourier spectrometer, obtaining its broadband reflectance characteristics. Additionally, the results are shown in Figure 2. It can be seen from the figure that when the wavelength was less than 120 μm, the reflectance of the sample remained at a low level, and the reflectance value was less than 0.1. When the wavelength is greater than 120 μm, the reflectance increased with the increase in the wavelength, and when the wavelength was around 300 μm, the reflectance was about 0.45.

The attenuation of the incident light by a black coating is exponentially related to the reciprocal of the wavelength [27,31]. For the coatings developed for the VIS and IR spectrum, as the wavelength increased, the absorption and scattering of the coating decreased, and the coating became gradually transparent so that the incident light could pass through the coating and be reflected by the metal substrate. Therefore, there is an increase in reflectivity.

Due to the increased reflectivity of the black paint surface, its diffuse and specular reflectance also have a higher value. Making the PST curve of the terahertz channel show strong prominent peaks at certain angles. The focus of stray light suppression in the THz channel should be to block the light outside the field of view from directly shining on the inner wall of the machine, which requires a combination of various measures to achieve the suppression of stray light at all off-axis angles.

### 3.2. Stray Light Suppression Structure

#### 3.2.1. Front Baffle Design

The front baffle is used to block stray light outside the field of view and is the first barrier for stray light suppression. The longer the front baffle, the smaller the shading angle, and the more effective it is to block external stray light. However, the length of the front baffle should also consider the actual load volume limit. The length of the front baffle is set to be equivalent to the length of the optical path. The outer envelope of the light entrance of the entire system is determined by the optical path of the THz channel. Therefore, the size of the front baffle is designed according to the optical path structure of the THz channel. The field of view of the system is a bar-shaped structure, and the front baffle is also set in a bar-shaped structure according to the range of the field of view. The cross-track field of view is 13°, which is larger than the along-track field of view. The position of the baffle vane is determined according to the cross-track field of view, which can make the baffle vane denser.

The length of the front baffle can be calculated as

(4) $$L=\frac{D_{0}}{\tan\varphi -\tan\omega },$$

where *L* is the length of the front baffle, set to 810 mm, which is equivalent to the length of the optical path, $D_{0}$ is the diameter of the light entrance, set to 730 mm, and φ and ω are the angle of light avoidance and the field of view, respectively. The field of view is ± 6.5°, so the angle of light avoidance is about 38°.

**Figure 3:**
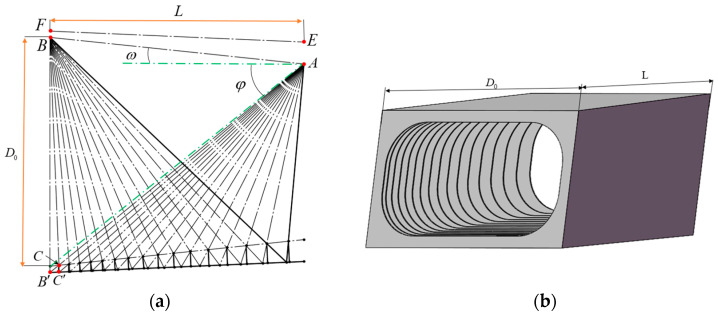
Design result of the front baffle. (**a**) Design principle of the baffle vane; (**b**) structure model of the front baffle.

The goal of the baffle vane is to make the incident stray light reach the image plane after at least two scattering in its interior [32]. The location of the front baffle vane can be determined as shown in Figure 3a: EF is the outer edge of the front baffle, and AB is the inner edge of the front baffle, that is, the edge of the field of view. Firstly, by connecting AB’, cross the edge of the field of view at point C, the position of the first baffle vane is obtained. Afterward, BC can be connected and extended to intersect the down outer edge of the front baffle at point C’, connecting AC’ again, and the intersection point with the down outer edge of the front baffle is the new position of the baffle vane. This step can be repeated to complete the final determination of the positions of all baffle vanes. The final front baffle model is shown in Figure 3b.

#### 3.2.2. Baffle between the Primary Mirror and the Third Mirror

**Figure 4:**
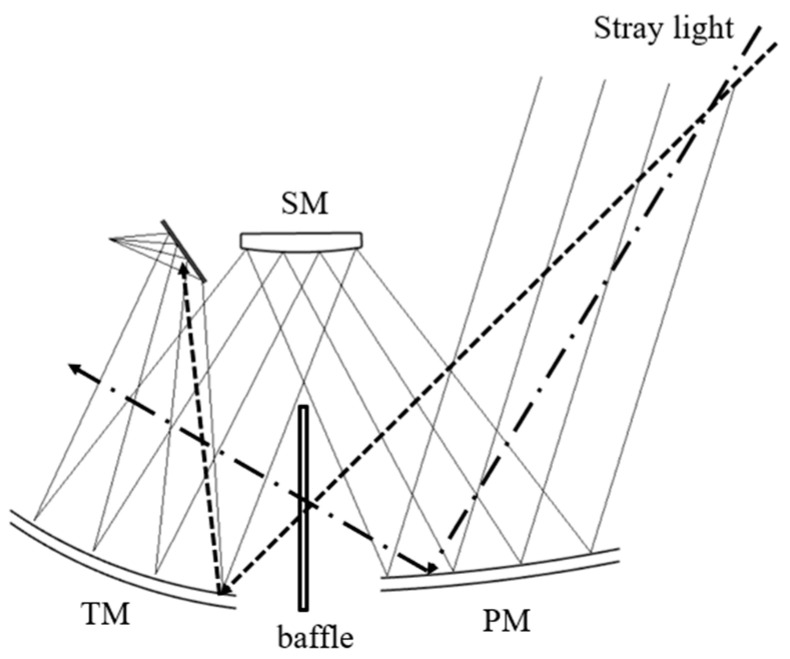
Stray light directly entering the interior.

Although the front baffle can effectively block the entry of external stray light, there are still some rays that directly illuminate the third mirror (TM) without passing through the primary mirror (PM) or are directly reflected into the interior by the PM. This light can be directly irradiated into the interior of the system, or even directly reflected by the TM into the THz detector, which is harmful to the imaging quality, as it shown in Figure 4. This part of the light can be blocked by setting a baffle between the PM and TM.

**Figure 5:**
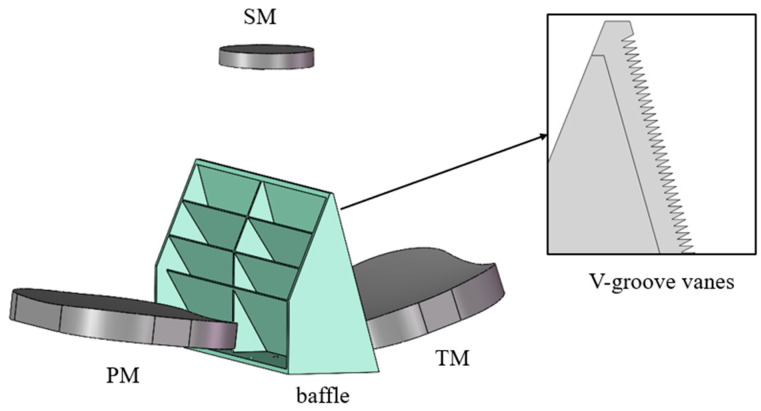
The baffle between PM and TM.

For the VIS and IR channels, setting the baffle can already effectively block the entry of this part of stray light. However, from the measurement results in Section 3.1, it can be seen that the reflectivity of black paint in the terahertz band is pretty high. Although the baffles installed between the PM and TM can effectively prevent the light from directly shining on the TM, there is still a part of the light scattered by the baffles and then transmitted to the secondary mirrors (SM), which perform TM reflection and finally enter the THz detector. Although this part of the light is scattered light, the reflectivity of the baffle, especially the SM and the TM, is very high, which eventually causes more serious stray light pollution. In order to solve this problem, a number of inclined side plates were added to the front of the baffle between the PM and TM, and the side plate tilts to a certain angle with the baffle to prevent the light that is scattered into the SM by the baffle or reflected into the PM and then into the SM. The incident light in this angle range is reflected or scattered by the side plates multiple times, which effectively reduces the energy reaching the THz focal plane. The baffle structure is shown in Figure 5.

#### 3.2.3. Reflective Inner Baffle

After passing through the front baffle and the baffle between the PM and TM, the system has a certain ability to suppress stray light, but it is still inevitable that the light of certain incident angles enters into the system, and this part of the light will still be reflected by the inner wall of the machine, and be transmitted to the THz focal plane. Further actions are needed. Stray light reaching the THz focal plane can be further reduced by placing an inner baffle in front of the focal plane.

**Figure 6:**
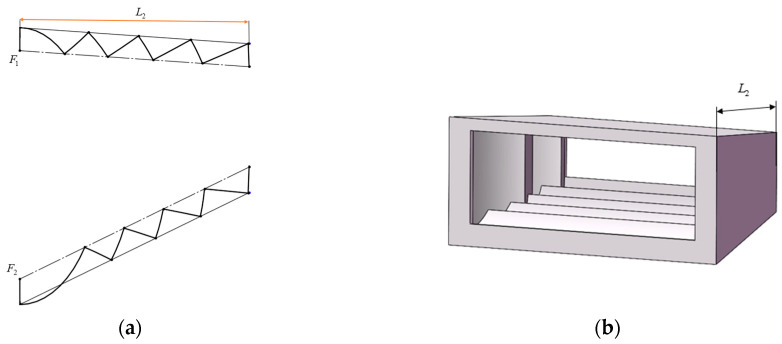
Design result of the inner baffle. (**a**) Design principle of the inner baffle; (**b**) structure model of the inner baffle.

The inner baffle adopts a reflective structure. The reason why the reflective structure is adopted is to reduce the emissivity of the inner baffle and reduce the influence of its own background radiation. The baffle adopts the Stavroudis type, which is composed of a confocal ellipsoid and a hyperboloid, the two are connected alternately, and all focal points are at the edge of the field of view at the entrance of the baffle [33]. Figure 6a is a schematic diagram of the two-dimensional structure of the baffle, the dotted line is the edge of the field of view, F_1_ and F_2_ are the focal points of the ellipse and the hyperbola, respectively, and Figure 6b is a model diagram of the inner baffle. Setting the length of the inner baffle to 40 mm, which is limited by the distance from the folding mirror to the focal plane, the size of the light inlet and outlet can be determined according to the light volume.

#### 3.2.4. Other Stray Light Suppression Measures

From the analysis results, when the off-axis angle range outside the field of view is small, the main source of stray light is the scattering or reflection of the frame around the SM; after that, it is the light baffle between the SM and the TM; with the off-axis angle appearing larger, and the top of the front baffle is a major source of stray light.

When the off-axis angle is small, part of the light shines on the periphery of the SM, and this light can be directly reflected or scattered by the peripheral bracket of the SM, or reflected by the upper half of the rear surface of the baffle between PM and TM and enter the THz detector. Stray light in this angular range can be reduced by placing the baffle or V-groove vanes structures that block light or scatter light that hits these surfaces in other directions. A similar approach can be taken for other critical surfaces as well.

## 4. Simulation Analysis

### 4.1. Analysis Model

**Figure 7:**
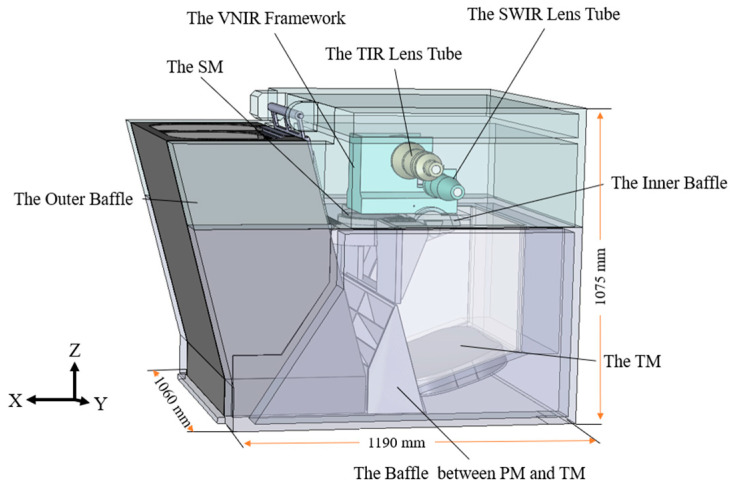
Optical machine structure mode.

According to the optical path structure and stray light suppression measures, an optical-mechanical model could be established and imported into stray light analysis software for stray light analysis. The model structure is shown in Figure 7. Using the ABg model to describe the bidirectional scattering distribution function (BSDF) of each surface. The calculation formula of the ABg model is [34]:

(5) $$BSDF(\left|\beta -\beta_{0}\right|)=\frac{A}{B+{\left|\beta -\beta_{0}\right|}^{g}},$$

where: β and $\beta_{0}$ are the projections of the scattering direction unit vector and the specular reflection direction unit vector on the surface, respectively. *A*, *B*, and *g* are the fitting coefficients.

The parameters of each surface of the system were set as follows: the absorptivity of the blackened coating was 0.94, 0.9, and 0.5 in the VNIR, TIR, and THz, respectively, and the scattering type was Lambertian scattering; The reflectivity of the mirror was set to 0.96 in the VNIR and SWIR bands, and 0.98 in the TIR and THz bands; the surface of the lens was coated with an anti-reflection coating; the surface of the SWIR lens was A = 10^−5^, B = 0.015, g = 2; the surface of the TIR lens was A = 0.00796, B = 0.01, g = 1.

### 4.2. External Stray Light Analysis

Point source transmittance (PST) can be used as the evaluation standard of the stray light suppression ability [35]. Point source transmittance only indicates the ability of the system itself to suppress stray light, and has nothing to do with the intensity of the light source outside the field of view, which can be defined as

(6) $$PST=\frac{E_{d}(\theta )}{E_{i}(\theta )},$$

where: *PST* is the point source transmittance, $E_{d}(\theta )$ is the irradiance of a point stray light source with an off-axis angle θ outside the field of view on the detector after passing through the optical system; $E_{i}(\theta )$ is the irradiance of the light source at the light entrance of the optical system.

**Figure 8:**
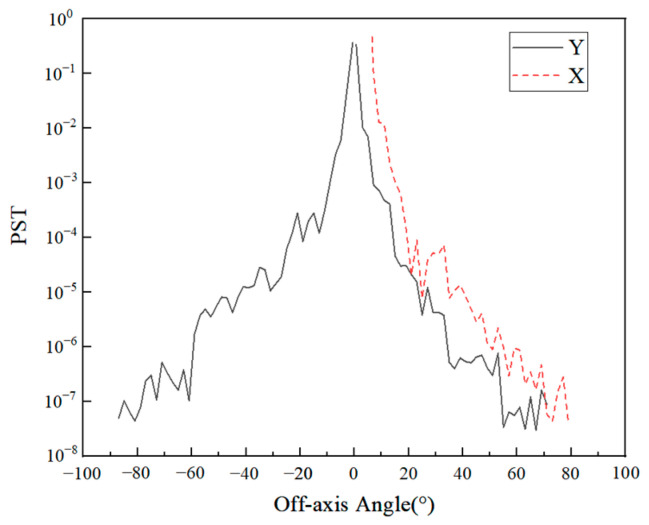
PST curves of the THz channel.

**Figure 9:**
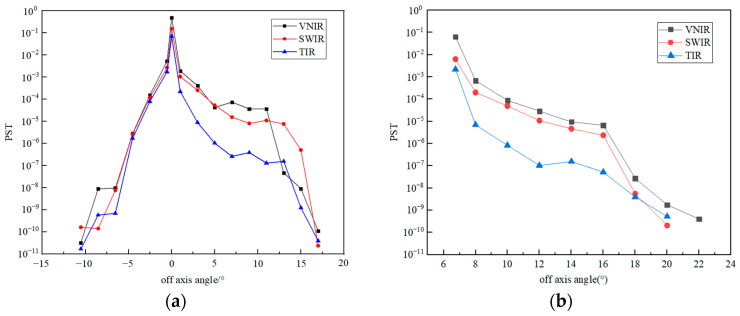
PST curves of VNIR, SWIR, and TIR channels. (**a**) Y direction; (**b**) X direction.

Ray tracing on the off-axis field of view of each channel can be performed, and the stray light PST curves can be obtained at different off-axis angles outside the field of view, as shown in Figure 8 and Figure 9. The system has symmetry in the X direction; the stray light effect caused by the +X direction and the −X direction is the same. Therefore, only a one-dimensional off-axis angle can be simulated in the X direction. During the simulation, for the Y direction, the X direction is fixed as the center of the observation field, and the same is true for the X direction.

The overall PST curve of the THz channel showed a downward trend, with no prominent peaks at special angles. After 10° off-axis, the decline was obvious, and then the decline was relatively slow. The final PST curve value was about an order of 10^−7^, and the overall stray light suppression effect was relatively good.

For VNIR, SWIR, and TIR channels, since the front-end system adopted a series of stray light suppression measures, the stray light entering the rear optical path was already very little. Therefore, ideal stray light suppression measures could be achieved only by setting the field stop near the primary image plane. For the −Y direction, the PST value dropped rapidly, and after only about 10° off-axis, its value dropped to the order of 10^−10^. For the +Y and +X directions, this decline was not as rapid as in the −Y direction due to a small amount of light that hits the inner wall of the system, but after an off-axis of no more than 20°, the PST value was also reduced to the order of 10^−10^. This shows that the front-end system and the field stop effectively blocked the entry of stray light.

### 4.3. Internal Stray Light Analysis

For the analysis of internal stray light, the operating temperature of the system was set to 293 K, and the operating temperature from the TIR detector to the cold stop was set to 77 K. The emissivity of each mirror was set to 0.01; the TIR emissivity of the blackened coating was 0.9, and the THz emissivity of the blackened coating was 0.5; the emissivity of the brightened surface was set to 0.03; the emissivity of the lens material Ge was 0.03, and the ZnSe was 0.0005 [36].

The TIR and THz signals observed by the instrument were the upward radiation from the earth or clouds, and finally reach the detectors. Its spectral radiance output $M_{\lambda }$ could be obtained by Planck’s law, and its expression is:

(7) $$M_{\lambda }=\frac{c_{1}}{\lambda^{5}}\frac{1}{e^{c_{2}/\lambda T}-1},$$

Among them, the first radiation constant is $c_{1}=3.7145\times 10^{4}\;W\cdot {\mathrm{cm}}^{-2}\cdot {\mu m}^{4}$, the second radiation constant is $c_{2}=1.43879\times 10^{4}\;\mu m\cdot K$, λ is the detection wavelength, and *T* is the target temperature.

The irradiance of the target signal finally reaching the detector could be expressed as

(8) $$E=\frac{\tau \int_{\lambda_{2}}^{\lambda_{1}}M(\lambda ,T)d\lambda }{4F^{2}},$$

where τ is the transmittance of the system, and *F* is the F number of the system. The F number is defined as the ratio of the system’s focal length f to the optical aperture D, that is,

(9) $$F=\frac{f}{D},$$

Based on this, the irradiance of the target signal finally reaching the detector can be estimated.

#### 4.3.1. Analysis of Terahertz Internal Stray Light

The emissivity of each surface is set as a surface light source, and the emission form is set as gray body emission. Ray tracing is performed on each surface in turn to obtain the irradiance reaching the detector. Finally, the stray irradiance reaching the detector can be obtained as shown in Table 2.

The signal radiated from the mechanical inner wall near the TM can easily enter the THz detector through the reflection of the TM and the scattering of other side walls, making the contribution of this part the largest.

**Figure 10:**
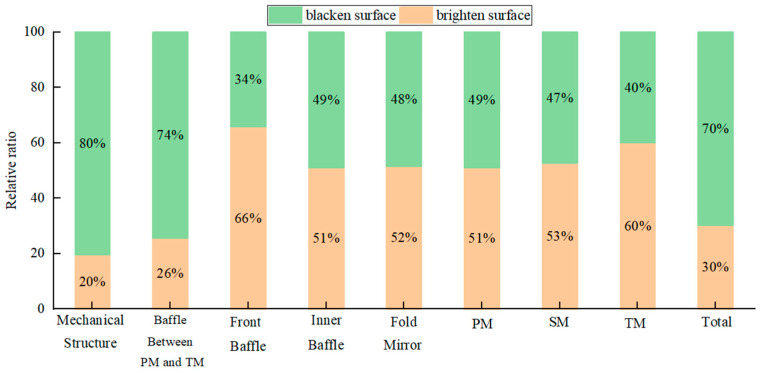
The relative ratio of brightened surface to blackened surface.

In order to reduce the influence of the system’s background radiation, the inner wall surface of the machine could be set as a brightener to reduce its surface emissivity [37]. When the inner wall of the machine was brightened, compared with the blackened surface, the irradiance reaching the THz detector dropped significantly. However, at the same time, due to the increase in reflectivity, the irradiance of other components reaching the detector increased, as shown in Figure 10. In general, the irradiance received by the detector was reduced to 2.02 × 10^−3^ W/m^2^, which was about 43% of the blackened structure.

The observation method in the terahertz channel is that the lower atmosphere radiates upward terahertz waves. Under clear sky conditions, the brightness temperature is generally 250 K, and the calculated irradiance on the image plane is about 1 × 10^−3^ W/m^2^. In this case, the background radiation is of the same order of magnitude as the target radiation and slightly larger than the target signal.

Although the use of surface brightness can effectively reduce the background radiation reaching the detector, the energy of the detection target is low, and further actions need to be taken to improve the overall sensitivity. For the background radiation flux, when the temperature control of the instrument is normal, the flux inside the instrument radiation should be stable. Therefore, the background signal can be monitored by setting the reference pixel, which is not used for scene imaging; only the background signal is received and is then subtracted from the imaging area signal [38,39].

The THz channel has fewer pixels and is a 10-element line array. The same element reference detector can be set parallel to the focal plane above and below the focal plane. The upper and lower reference pixel signals of each imaging pixel can be averaged as the background signal of the pixel. The background irradiance of the instrument reaching each pixel was simulated under 293 K. The average irradiance reaching the upper and lower reference pixels was 1.92 × 10^−3^ W/m^2^, which is slightly different from the 2.02 × 10^−3^ W/m^2^ irradiance reaching the focal plane.

**Figure 11:**
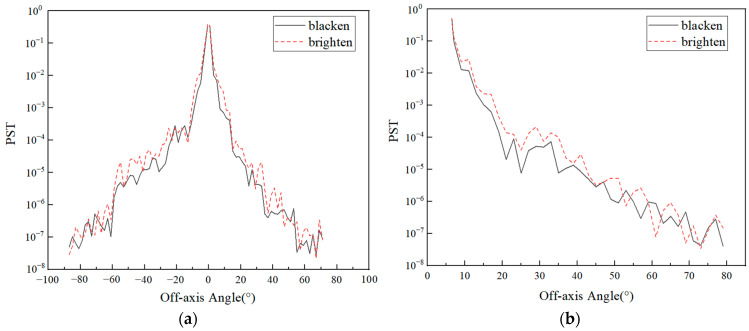
PST curves of THz with inner wall brighten or blacken. (**a**) Y direction; (**b**) X direction.

In addition, the use of a total reflection structure on the inner wall increases the reflectivity, which, in turn, leads to an increase in external stray light. The simulation of the external stray light of the system with the inner wall surface brightened can be carried out again, and the result is shown in Figure 11. 

On the whole, compared with the blackening of the inner surface, the PST curve of the brightened surface structure has little difference, but there is a certain uplift in some angles. This is because the front baffle and the baffle between PM and TM have good shielding effect on stray light at most separation axis angles. However, there is still a part of the angle light that could enter the inner wall. When the inner wall reflectivity increases, the energy reaching the detector increases. However, as a balance between the contribution of internal and external stray light, it is a good choice to set the inner wall near the TM brightened. Although this increases the energy of external stray light at some angles, it can effectively reduce internal stray light. Additionally, it has little effect on the VIS and IR channels.

#### 4.3.2. Analysis of the Thermal Infrared Channel Internal Stray Light

Using the same method, the simulation analysis of the TIR internal stray light was carried out, and the results are shown in Table 3.

In the whole system, the lens tube of the rear optical path contributes the most, accounting for 67.85%. In addition, the mechanical surface also has a certain contribution. The final background radiation contribution can be reduced by setting the inner surface of the lens tube to brighten and using a black nickel structure on the key surface of the mechanical structure.

The modified simulation results can be compared to the blackening of the lens tube surface. After the surface emissivity is optimized, the background irradiance received by the image plane of the entire system is reduced to 0.172 W/m^2^, which is about 1/2.3 of the original. The simulation results of the external stray light showed that compared with the blackened structure of the lens tube, the external stray light barely changed.

**Figure 12:**
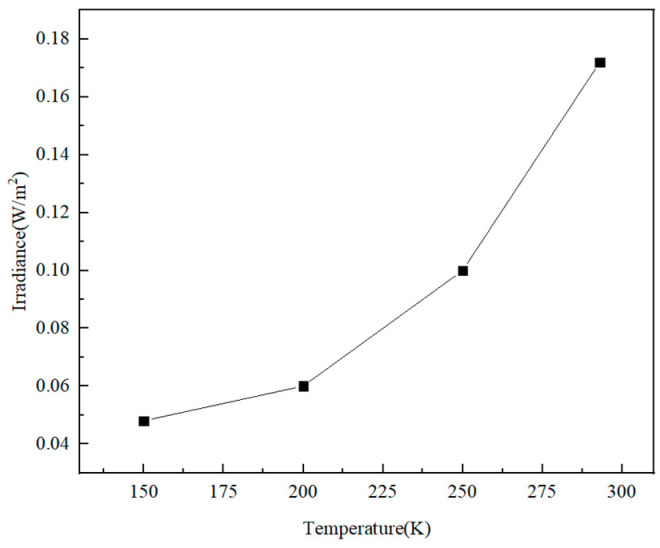
The stray light irradiance of the image plane at different rear optical path temperatures.

Using Formula (8), the irradiance of the 250 K infrared target arriving at the detector pixel is 0.47 W/m^2^, the irradiance of the target signal is higher than that of the stray signal, and the signal-to-noise ratio is 2.73. In addition, the background radiation of the system can be further reduced by cooling the rear optical path. The internal stray light received by the image plane at different rear optical path temperatures is shown in Figure 12. When the temperature of the rear optical path is reduced to 150 K, the signal-to-noise ratio can be increased by about 10 times.

Through the optimization of the surface emissivity of the key surface and partial temperature control, the internal radiation of the system can be controlled in a lower range at a lower cost.

## 5. Conclusions

In this study, according to the wide detection wavelength characteristics of the visible to terahertz integrated cloud detection imaging system, combined with the structure of the optical system, stray light elimination measures such as the front baffle and the baffle between PM and TM, were designed. According to the characteristics of the surface material at the terahertz wavelength, special settings were made for the baffles between PM and TM, and a reflective inner baffle was set. The simulation results showed that the stray light suppression effect of the visible infrared channel was very obvious. When the off-axis angle outside the field of view was greater than 20°, the PST value was less than 10^−7^. For the terahertz channel, when the angle was greater than 30°, the PST curve could reach the order of 10^−5^. At the same time, the internal stray characteristics of the terahertz and thermal infrared channels were analyzed, and the influence of internal stray light could be effectively reduced through reasonable surface properties and temperature control, and have less impact on external stray light. This research can provide a certain reference for the analysis and suppression of stray light based on conventional surface materials with a broad spectrum or terahertz optical systems.

## Figures and Tables

**Table 1:** Optical design parameters.

Parameter	Values
Channel Central wavelength (μm)	VNIR: 0.470, 0.550, 0.650, 0.865 SWIR: 1.640, 2.100 TIR: 8.500, 11.000, 12.000 THz: 343.25 (874 GHz)
Resolution(m) (@450 km orbit altitude)	VNIR: 75 SWIR, TIR: 100 THz: 10,000
Swath width (km)	100

**Table 2:** Irradiance received by the THz detector.

Stray Light Source	Irradiance (W/m^2^)	Percentage (%)
Mechanical Structure	3.37 × 10^−3^	72.48
Baffle between PM and TM	3.07 × 10^−4^	6.60
Front Baffle	6.81 × 10^−5^	1.47
Inner Baffle	7.40 × 10^−4^	15.93
Fold Mirror	7.95 × 10^−5^	1.71
PM	1.80 × 10^−5^	0.39
SM	1.91 × 10^−5^	0.41
TM	4.70 × 10^−5^	1.01
Total	4.64 × 10^−3^	100.00

**Table 3:** Irradiance received by the TIR detector.

Stray Light Source	Irradiance/(W·m^−2^)	Percentage/%
Mechanical Structure	2.13 × 10^−2^	5.48
Fold Mirror of TIR	5.39 × 10^−3^	1.38
Lens Tube	2.64 × 10^−1^	67.85
Dichroics	8.58 × 10^−3^	2.21
Lens 1	4.68 × 10^−4^	0.12
Lens 2	3.59 × 10^−4^	0.09
Lens 3	3.85 × 10^−2^	9.90
Lens 4	5.02 × 10^−2^	12.91
Others	2.36 × 10^−4^	0.06
Total	3.89 × 10^−1^	100.00

## Data Availability

Not applicable.

## References

[1] Shang H.Z., Husi L.T., Li M., Tao J.H., Chen L.F. (2022). Remote Sensing of Cloud Properties Based on Visible-to-Infrared Channel Observation from Passive Remote Sensing Satellites. Acta Opt. Sin..

[2] Bony S., Stevens B., Frierson D.M.W., Jakob C., Kageyama M., Pincus R., Shepherd T.G., Sherwood S.C., Siebesma A.P., Sobel A.H. (2015). Clouds, Circulation and Climate Sensitivity. Nature Geosci..

[3] Schueler C., Clement J.E., Ardanuy P.E., Welsch M.C., DeLuccia F., Swenson H. (2002). NPOESS VIIRS Sensor Design Overview. Proceedings of the Earth Observing Systems VI.

[4] Schmülling F., Zerfowski I., Pillukat A., Bonsignori R. (2010). METimage: A multispectral imaging radiometer for the EUMETSAT Polar System follow-on satellite mission. Proceedings of the Sensors, Systems, and Next-Generation Satellites XIV.

[5] Hu X.Q., Xu H.L., Lei S.T., Wang L., Yu T.L., Wang Y., Gao Y., Hu S.S., Xu N., Chen L. (2022). Overview of Low Light Detection and Application of FY-3 Early Morning Satellite. Acta Opt. Sin..

[6] Buehler S.A., Jiménez C., Evans K.F., Eriksson P., Rydberg B., Heymsfield A.J., Stubenrauch C.J., Lohmann U., Emde C., John V.O. (2007). A concept for a satellite mission to measure cloud ice water path, ice particle size, and cloud altitude. Q. J. R. Meteorol. Soc..

[7] Fox S. (2020). An Evaluation of Radiative Transfer Simulations of Cloudy Scenes from a Numerical Weather Prediction Model at Sub-Millimetre Frequencies Using Airborne Observations. Remote Sens..

[8] Bergadá M., Labriola M., Gonzalez R., Palacios M.A., Marote D., Andrés A., García J.L., Sánchez-Pascuala D., Ordóñez L., Rodríguez M. The Ice Cloud Imager (ICI) preliminary design and performance. Proceedings of the 2016 14th Specialist Meeting on Microwave Radiometry and Remote Sensing of the Environment (MicroRad).

[9] Waters J.W., Froidevaux L., Harwood R.S., Jarnot R.F., Pickett H.M., Read W.G., Siegel P.H., Cofield R.E., Filipiak M.J., Flower D. (2006). The earth observing system microwave limb sounder (EOS MLS) on the aura satellite. IEEE Trans. Geosci. Remote Sens..

[10] Paige D.A., Foote M.C., Greenhagen B.T., Schofield J.T., Calcutt S., Vasavada A.R., Preston D.J., Taylor F.W., Allen C.C., Snook K.J. (2010). The Lunar Reconnaissance Orbiter Diviner Lunar Radiometer Experiment. Space Sci. Rev..

[11] McCleese D.J., Schofield J.T., Taylor F.W., Calcutt S.B., Foote M.C., Kass D.M., Leovy C.B., Paige D.A., Read P.L., Zurek R.W. (2007). Mars Climate Sounder: An investigation of thermal and water vapor structure, dust and condensate distributions in the atmosphere, and energy balance of the polar regions. J. Geophys. Res. Planets.

[12] Li J., Yang Y., Qu X., Jiang C. (2022). Stray Light Analysis and Elimination of an Optical System Based on the Structural Optimization Design of an Airborne Camera. Appl. Sci..

[13] Wei L., Yang L., Fan Y.-P., Cong S.-S., Wang Y.-S. (2022). Research on Stray-Light Suppression Method for Large Off-Axis Three-Mirror Anastigmatic Space Camera. Sensors.

[14] Pilbratt G.L., Riedinger J.R., Passvoge T., Crone G., Doyle D., Gageur U., Heras A.M., Jewell C., Metcalfe L., Ott S. (2010). Herschel Space Observatory—An ESA facility for far-infrared and submillimetre astronomy. Astron. Astrophys..

[15] Fu Y. (2014). Cloud parameters retrieved by the bispectral reflectance algorithm and associated applications. J. Meteorol. Res..

[16] Ackerman S.A., Smith W.L., Revercomb H.E. (1990). The 27–28 October 1986 FIRE IFO cirrus case study: Spectral properties of cirrus clouds in the 8–12 μm window. Mon. Weather. Rev..

[17] Buehler S.A., Defer E., Evans K.F., Eliasson S., Mendrok J., Eriksson P., Lee C., Jimenez C., Prigent C., Crewell S. (2012). Observing ice clouds in the submillimeter spectral range: The CloudIce mission proposal for ESA’s Earth Explorer 8. Atmos. Meas. Tech. Discuss..

[18] Liu L., Weng C., Li S., Husi L., Hu S., Dong P. (2021). Passive Remote Sensing of Ice Cloud Properties at Terahertz Wavelengths Based on Genetic Algorithm. Remote Sens..

[19] Dong P., Liu L., Li S., Hu S., Bu L. (2021). Application of M5 Model Tree in Passive Remote Sensing of Thin Ice Cloud Microphysical Properties in Terahertz Region. Remote Sens..

[20] Dubreuil D., Martignac J., Toussaint J.C., Visticot F., Delisle C., Gallais P., Pennec J.L., Lerch T., André P., Lortholary M. (2014). Optical Design for the 450, 350, and 200 μm ArTeMiS Camera. Proceedings of the Millimeter, Submillimeter, and Far-Infrared Detectors and Instrumentation for Astronomy VII.

[21] Jiang H.W., Niu X.H. (2023). Design of Integrated Cloud Detection Optical System from Visible to Terahertz Bands. Acta Opt. Sin..

[22] Jiang S.W., Xia Z.T., Sun Y.X., Wang K. (2020). Optical Design and Stray-Light Analysis of Urban Night-Light Remote Sensing Imaging System. Laser Optoelectron. Prog..

[23] Wang H., Cheng Q.F., Ma Z.P., Yan H.L., Lin S.M. (2022). Development and Prospect of Stray Light Suppression and Evaluation Technology(Invited). Acta Photonica Sin..

[24] Clermont L., Aballea L. (2021). Stray light control and analysis for an off-axis three-mirror anastigmat telescope. Opt. Eng..

[25] Xue Q.S. (2016). Optical Design and Stray Light Analysis for Large Aperture Catadioptricstar Sensor. Acta Opt. Sin..

[26] Zhou J., Li J., Wang Q.F., Xu M.D., Zhang C.Y. (2015). Optimized Design of Infrared Opto-Mechanical Systems Based on the Spontaneous Emission Suppression. Acta Opt. Sin..

[27] Smith S.M. (1984). Specular reflectance of optical-black coatings in the far infrared. Appl. Opt..

[28] Shi J., Zhong K., Liu C., Wang M.R., Qiao H.Z., Li J.N., Xu D.G., Yao J.K. (2018). Scattering properties of rough metal surface in terahertz region. Infrared Laser Eng..

[29] Blue M.D., Perkowitz S. (1992). Space-exposure effects on optical-baffle coatings at far-infrared wavelengths. Appl. Opt..

[30] Fang B., Qi C.K., Deng Y.Q., Gao Y.J., Cao J.H., Yao Z.G., Xia M.R. (2019). Characteristics of Highly Absorptive Coatings Used in Terahertz Radiometry. Chin. J. Lasers.

[31] Monte C., Gutschwager B., Adibekyan A., Hollandt J. (2014). A Terahertz Blackbody Radiation Standard Based on Emissivity Measurements and a Monte-Carlo Simulation. J. Infrared Millim. Terahertz Waves.

[32] Chen X., Hu C.H., Yan C.X., Kong D.C. (2019). Analysis and suppression of space stray light of visible cameras with wide field of view. Chin. Opt..

[33] Fest E.C. (2013). Stray Light Analysis and Control.

[34] Freniere E.R., Gregory G.G., Chase R.C. (1997). Interactive software for optomechanical modeling. J. Roy Anthr. Inst..

[35] Zhang Z.N., Li L.B., Zhou C.B., Hao X.B., Sun J., Liu X.B., Wang P.C., Liu J. (2021). Stray Light Suppression in Sweep Mirror Field-Widene d Space-Borne Fourier Transform Imaging Spectrometer. Acta Opt. Sin..

[36] Zhao C.Y., Xu Y.J., Shao W., Zhang L.G., Ren J.Y. (2015). Stray Light Analyze and Suppress of the Space-Borne Infrared Optical System. Chin. J. Lasers.

[37] Xie X.L., Zhu X.X., Zhu J.C., Shen W.M. (2022). Analysis and suppression of stray radiation in uncooled thermal infrared imaging spectrometer. Acta Opt. Sin..

[38] Albiñana A.P., Gelsthorpe R., Lefebvre A., Sauer M., Weih E., Kruse K.-W., Münzenmayer R., Baister G., Chang M. (2010). The multi-spectral imager on board the EarthCARE spacecraft. Proceedings of the Infrared Remote Sensing and Instrumentation XVIII.

[39] Kato E., Katayama H., Naitoh M., Harada M., Nakamura R., Sakai M., Nakajima Y., Nakau K., Tange Y., Sato R. (2014). Radiometric calibration of Compact Infrared Camera (CIRC) for earth observation. Sens. Mater.

